# Prevalence and determinants of Soil-Transmitted Helminths among urban vegetable farmers in Ghana

**DOI:** 10.1371/journal.pone.0323486

**Published:** 2025-05-15

**Authors:** Gerard Quarcoo, Samuel Armoo, Augustina Angelina Sylverken, Matthew Glover Addo

**Affiliations:** 1 Environmental Biology, Biotechnology and Health Division, Council for Scientific and Industrial Research, Water Research Institute, Achimota, Greater Accra, Ghana; 2 Department of Theoretical and Applied Biology, Kwame Nkrumah University of Science and Technology, PMB, KNUST, Kumasi, Ghana; 3 Biomedical and Public Health Research Unit, Council for Scientific and Industrial Research-Water Research Institute, Achimota, Greater Accra, Ghana; 4 Kumasi Centre for Collaborative Research in Tropical Medicine, Kwame Nkrumah University of Science and Technology, PMB, KNUST, Kumasi, Ghana; Quinnipiac University, UNITED STATES OF AMERICA

## Abstract

**Introduction:**

Urban vegetable farmers in Ghana face multiple health risks, including soil-transmitted helminths (STHs), which may contribute to morbidities that threaten urban food security. Additionally, infected farmers may act as persistent sources of disease transmission within urban populations. There is the need to assess the burden of STH among these farmers using more sensitive molecular assays.

**Methods:**

This was a cross-sectional study involving 168 urban farmers from Accra and Tamale in Ghana’s Greater Accra and Northern regions, respectively. Participants completed semi-structured questionnaires, and stool samples were collected for analysis. A qualitative Polymerase Chain Reaction (QPCR) assay was employed to detect STH prevalence, targeting the *ITS1, ITS2,* and *18S* genes of *Ascaris lumbricoides*, *Ancylostoma duodenale* and *Strongyloides stercoralis*, respectively.

**Results:**

While no positives were found in Tamale, 5.1%, 2.5%, and 0.8% of participants in Accra tested positive for *A. lumbricoides*, *A. duodenale* and *S. stercoralis*, respectively. Inadequate use of Personal Protective Equipment (PPEs) and STH infection status were strongly correlated among risk factors (Odds ratio; 4.3, 95% Cl: 1.03–18.00, *p–value = *0.04). Overall, 72% of participants in Tamale wore PPEs, compared to 43% in Accra.

**Conclusions:**

Even though STH was not common, inadequate PPE use was a major factor in STH transmission in urban vegetable farms. Therefore, the key to drastically lowering the STH burden in urban farms is education and behaviour changes. Using more sensitive molecular diagnostic assays is crucial in low prevalence environments.

## 1. Introduction

Soil-transmitted helminths (STHs) remain a global health burden, being the most prevalent of the Neglected Tropical Diseases (NTDs) [1]. These STHs are parasitic helminths that inhabit the intestine of humans resulting in a range of associated morbidities. Even though the burden of STHs has decreased as a result of interventions, 1.5 billion individuals globally are still impacted by STHs [2]. *Strongyloides stercoralis*, roundworms (*Ascaris lumbricoides*), whipworms (*Trichuris trichiura*), and hookworms (*Ancylostoma duodenale, Necator americanus*) are four parasitic helminths that are mostly responsible for STH infections and morbidities worldwide [3]. Risk factors such as inadequate sanitation practices are crucial for the transmission of STHs [4,5], therefore the majority of STH infections are found in impoverished settlements within 94 countries in Africa, Latin America and Asia [6,7].

The urban food value chain relies heavily on urban farmers. Urban farmers’ efforts contribute to the achievement of Sustainable Development Goals (SDG) 2, which is to eradicate hunger among urban dwellers [8]. Urban farmers contributed to the provision and upkeep of food security in urban areas during the recent COVID-19 pandemic, and the associated lockdowns in numerous cities worldwide, saving lives. These urban farmers, particularly those in lower middle-income countries like Ghana, are exposed to multiple health risks including the risk of contracting STH infections [9] due to the possibility that the eggs of STHs found in the stool of infected people could contaminate soils and irrigating water sources. Transmission of STHs occur when individuals get into direct contact with these contaminated soil and water sources, and the majority of urban farmers in Ghana use contaminated water from open drains, streams and ponds [10–12].

Despite the high risk of STH infection faced by urban farmers, most studies on STH in Ghana have largely focused on pregnant women and children [13]. On environmental matrixes, STHs have been detected in soils, water used for irrigation, irrigated vegetables, sludge and ready-to-eat foods sold for human consumption [14,15], but scarcely in the urban vegetable farmers. As far as we know from our PubMed searches, only one study has explored the prevalence of STH among urban vegetable farmers in Ghana [16] which was undertaken in a single location and used microscopy for STH detection. This, however, does not offer a wider representation of the STH prevalence among urban vegetable farmers in Ghana. Moreover, the diagnostic specificity and sensitivity of microscopy are limited in low prevalence settings, which may result in an underestimation or overestimation of STH intensity [17,18] There have been major interventions against STHs, including school-based mass administration of anthelminthics [19] and improved access to over-the-counter anthelminthics for both adults and children. It is therefore important to continually use highly sensitive and specific approaches to investigate the burden of STHs among at-risk groups such as urban vegetable farmers to help assess the impact of these interventions. This study, therefore aimed to determine the prevalence of STHs among urban vegetable farmers from multiple locations in two different regions of Ghana (Northern and Greater Accra regions) employing the use of a molecular technique - qualitative polymerase chain reaction (QPCR).

## 2. Materials and methods

This was a cross-sectional study that involved administration of questionnaire on the field as well as laboratory analysis of stool samples collected from study participants.

### 2.1 Study area

**Fig 1:**
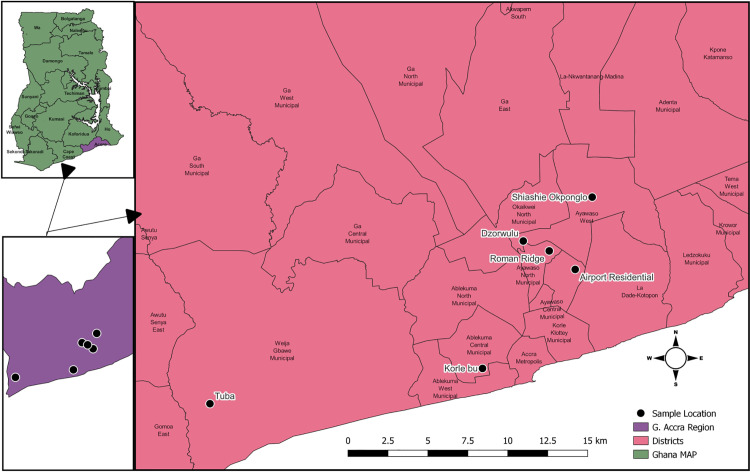
Stool sampling sites in Greater Accra, Ghana (Source: Field data collection).

**Fig 2:**
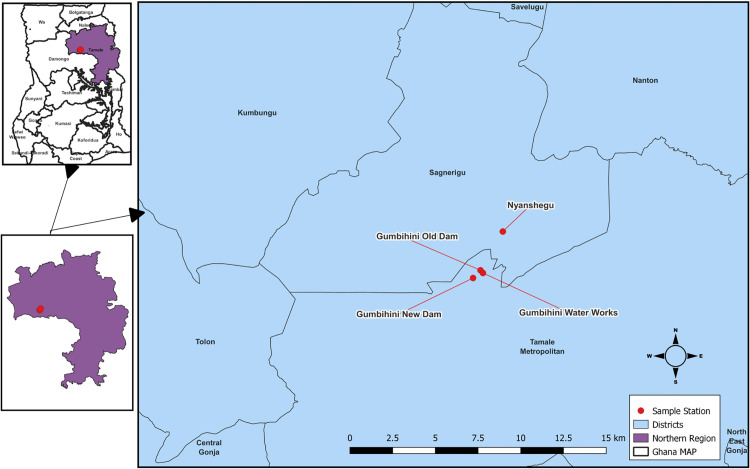
Stool sampling sites in Tamale, Ghana (Source: Field data collection).

This study focused on urban vegetable farmers from the Accra and Tamale Metropolitan areas, located in the Greater Accra and Northern regions of Ghana, respectively. The study areas primarily rely on wastewater, mostly untreated and sourced from open drains, for irrigating vegetable crops [15,20]. This practise poses a significant risk factor for helminth infections among these farmers [16,21]. Other available irrigating water sources included shallow ponds, dugouts and an occasional use of improved water sources. In Greater Accra, the vegetable farmers were enrolled from major vegetable farms such as Dzorwulu and environs, (longitude: -0.214619 latitude: 5.612327), Korle-Bu (longitude: -0.237643, latitude: 5.540294), and Tuba (longitude: -0.391613, latitude: 5.520356) vegetable farms (Fig 1). In Tamale, the farmers were selected from Gumbihini Old and New Dam (longitude: -0.847760, latitude: 9.416068), Gumbihini Waterworks (longitude: -0.846462, latitude: 9.414615) and Nyanshegu (longitude: -0.835872, latitude: 9.436735) vegetable farms (Fig 2)). The maps of the study sites are presented as supplementary materials. These sites contribute to the urban food baskets via the cultivation and provision of fresh vegetables such as lettuce, cabbage and spring onions in large quantities to major markets. Thereby, contributing to the achievement of food security and improvement in the nutritional well-being of the urban dwellers. All the vegetable farms in Greater Accra and Tamale had assigned Agric Extension Officers (AEOs) who visit and monitor the activities of these farmers [22].

### 2.2 Sample collection

Ethical clearance (reference number CHRPE/AP/645/19 renewed to CHRPE/AP/027/21) was obtained from the Committee on Human Research, Publication and Ethics of Kwame Nkrumah University of Science and Technology (CHRPE- KNUST). Study participants were selected through purposive sampling only after completing an informed consent process. Participants were selected based on the following criteria: a) being an adult above 18 years of age; b) having worked on vegetable farms for at least one year, and c) not having undergone any deworming treatment within four weeks prior to the data collection period. Confidentiality was guaranteed by assigning each participant a unique identification code, which was used to label the sterile stool containers for all consenting participants.

The participants were interviewed between 1^st^ to 4^th^ November 2020 and from 21^st^ January to 31^st^ October 2021 for Tamale (n = 54) and Greater Accra (n = 125), respectively. The semi-structured questionnaire solicited respondents’ socio-demographics and risk factor characteristics such as type of irrigating water sources, deworming exercises undertaken, personal protective equipment (PPE) usage and personal hygiene practices in the farming activities.

After questionnaire administration, the participants were asked to present the stool samples the next morning between 6 am to 9 am. The samples were then collected in a cooling box for parasitological analysis. To preserve the Tamale stool samples during the long travel time from the study sites to the laboratory, absolute ethanol was added to the collected samples [23] and preserved on ice. The samples were transported to the Biomedical and Public Health Unit of the Council for Scientific and Industrial Research – Water Research Institute, Accra for molecular analysis. From the total sample size calculation of 155 estimated from a previous study [16], 168 stool samples were collected out of the 179 urban vegetable farmers interviewed.

### 2.3 Laboratory procedures

DNA for detection of *A. duodenale, N. americanus*, *A. lumbricoides*, *T. trichiura* and *S. stercoralis* was extracted from the stool samples using an optimised protocol of Basuni et al. [24] with Quick Zymo DNA test kits in accordance with the manufacturer’s instructions. Before DNA extraction from the stool samples collected in Tamale, sterile distilled water was used to remove absolute ethanol used for preservation as done elsewhere [25,26]. Briefly, 0.1g of the stool sample was suspended in 500 μL of 2% Polyvinylpolypyrrolidone in Phosphate Buffered Saline (PVPP/PBS), seeded with glass beads and then vortexed for 10 s at 3000 rpm, and then incubated overnight at -20 ºC. The sample was defrosted and processed using bead beating with the MagNa Lyser (Roche Diagnostics GmbH, Mannhelm Germany), for 5 min at 3000 rpm. The suspension was treated 200 μL of the Genomic Lysis Buffer, to which 20 μL Proteinase K was added, and briefly vortexed. This was followed by a series of washes using a spin column, culminating in the final elution of DNA.

*A. duodenale, N. americanus*, *A. lumbricoides*, *T. trichiura* and *S. stercoralis* were then detected using optimised PCR assays of ten Hove et al., and Kaisar et al. [25,26]. For the PCR reaction mix had a total volume of 12 μL, constating of 5 µ L of SYBR Green Master Mix, 0.4 µ L of primers [24,18] (Table 1), 4.6 µ L of nuclease-free water, and 2 µ L of DNA template. Each run was performed on the Eppendorf Mastercycler (Hamberg, Germany) using the following reaction conditions: initial incubation at 95°C for 3 minutes, followed by 45 cycles of 54°C for 30 seconds, and 72°C for 30 seconds, and a final incubation step of 72°C for 4 minutes. PCR products were run on a 2% agarose gel, and visualised for gel documentation and DNA fragment characterization using UV illuminator (Benchtop Variable Transilluminator, Cambridge, UK).

**Table 1:** Primers for the detection of Soil-transmitting helminths and internal control virus.

Soil-transmitted helminths/ Target organism	Primer Name	Primer Sequence	Expected bp^*^	Reference
*A. lumbricoides*	*ALUM96F*	GTAATAGCAGTCGGCGGTTTCTT	89	[24]
	*ALUM183R*	GCCCAACATGCCACCTATTC		
*A. duodenale*	*AD125F*	GAATGACAGCAAACTCGTTGTTG	71	
	*AD195R*	ATACTAGCCACTGCCGAAACGT		
*S. stercoralis*	*STRO8S-153F*	GAATTCCAAGTAAAACGTAAGTCATTAGC	101	
	*STRO18S-1630R*	TGCCTCTGGATATTGCTCAGTTC		
*N. americanus*	*NA58 F*	CTGTTTGTCGAACGGTACTTGC	101	
	*NA158R*	ATAACAGCGTGCACATGTTGC		
*T. trichuria*	*TT185F*	TTGAAACGACTTGCTCATCAACTT	76	[18]
	*TTI85R*	CTGATTCTCCGTTAACCGTTGTC		
Phocine herpesvirus	*PhHV-267s*	GGGCGAATCACAGATTGAATC	69	[24]
	*PhHV-337as*	GCGGTTCCAAACGTACCAA		

*bp=base pair

### 2.4 Quality control

Nuclease free water was used as a negative control for all analyses. Positive reference controls were obtained from archival DNA of the individual parasites stored in the Biomedical and Public Health Unit of the Council for Scientific and Industrial Research – Water Research Institute. Internal process control for the DNA extraction was assessed using Phocine herpes virus (PhHV-1) [24]. PCR products with amplified gene targets of expected sizes 89, 71, 101, 101, 76, and 69 base pairs were deemed positive for *A. lumbricoides, A. duodenale, S. stercoralis*, *N. americanus, T. trichiura* and PhHV-1 respectively (Table 1) when compared with an ultra-low molecular gene marker (Quickload®, Biolabs United Kingdom).

### 2.5 Data management and statistical analysis

Results of the molecular parasitological assessments were recorded on proforma data sheets. The data were then double entered into Microsoft Excel prior to statistical analyses using the Statistical Package for Social Science (SPSS) software (IBM version 27.0: IBM Corp, Armonk, NY, USA). Microsoft Excel version (2206) was used to generate summary tables from the responses from the questionnaire administration. The binary logistic regression model was used to investigate the associations between risk factor characteristics solicited from the questionnaires and STH infection among these farmers. The Odds ratio (OR) analysis was used to determine risk factors affecting the occurrence of STH infections among vegetable farmers with a confidence interval (Cl) of 95%.

## 3. Results

### 3.1 Prevalence of STHs

Vegetable farmers located in Greater Accra tested positive for STHs, as amplification of ITS1 and ITS*2* genes confirmed the presence of roundworm (*A. lumbricoides*) and hookworm (*A. duodenale*) parasites, respectively. These STHs had the highest prevalence rates among farmers, with *A. lumbricoides* at 5.1% (6/118, 95% Cl = 2.1–11.2) and *A. duodenale* at 2.5% (3/118, 95% Cl = 0.7–7.8). Additionally, amplification of the *18S* gene (101 bp) indicated the presence of *S. stercoralis* at a prevalence of 0.8% (1/118, 95% Cl = 0.0–5.3).

In contrast, vegetable farmers in Tamale, located in the Northern region, showed no evidence of STH infections, as there was no amplification of their targeted genes in their stool samples (Table 2).

**Table 2:** Prevalence of STHs in stool samples collected from urban vegetable farmers in Northern (Tamale) and Greater Accra regions (November 2020 – October, 2021).

STHs Primer target	Tamale			Greater Accra		
	N = 50			N = 118		
	Infected	Prevalence	(95% Cl)	Infected	Prevalence	(95% Cl)
			(%)			(%)
*Ascaris lumbricoides*	0	0.0	–	6	5.1	2.1 - 11.2
*Ancylostoma duodenale*	0	0.0	–	3	2.5	0.7 - 7.8
*Strongyloides stercoralis*	0	0.0	–	1	0.8	0.0 - 5.3
*Necator americanus*	0	0.0	–	0	0.0	–
*Trichuris trichuria*	0	0.0	–	0	0.0	–

*N = the total number of participants*

*Prevalence (%) = Prevalence of positive outcome (*$Prev=\frac{n(positive)}{N}*100$)

*(95% Cl) = prevalence with 95% confidence interval*

### 3.2 Farmers’ demographics, irrigation water source and deworming practice

In Tamale, in the Northern region, the majority of the urban farmers (38.9%, 21/54) had no formal education, and most (29.6%, 16/54) were aged between 41–50 years. The study revealed that urban vegetable farming in the area was exclusively male (100%, 54/54). Among these farmers, the majority (72.2%, 39/54) reported using PPEs, such as boots and gloves while working on their farms and practiced hand washing with soap after work. However, a significant proportion (59.3%, 32/54) had not dewormed for the period of 12 -months and beyond. The primary irrigation water source used by these farmers was improved water sources (64.8%, 35/54) (Table 3).

**Table 3:** Descriptive summary of urban vegetable farmers in Tamale and Greater Accra demographic (November 2020 – October, 2021).

Variable	Variable description	Distribution of responses			
		Tamale (N = 54)		Accra (N = 125)	
		n	%	n	%
Gender	Male	54	100.0	123	98.0
	Female	0	0.0	2	1.6
Age	<20	0	0.0	8	6.4
	20-30 years	13	24.1	51	40.8
	31-40 years	14	25.9	28	22.4
	41–50 years	16	29.6	25	20.0
	51–60 years	3	5.6	6	4.8
	> 60 years	8	14.8	7	5.6
Education	No formal education	21	38.9	18	14.4
	Basic	10	18.5	68	54.4
	Senior High/A’ level	15	27.8	22	17.6
	Tertiary	2	3.7	8	6.4
	Informal education	6	11.1	3	2.4
	Others (Arabic school)	0	0.0	6	4.8
Irrigating water source	Improved water	35	64.8	27	21.6
	Surface water (dam, pond, drain)	19	35.2	67	53.6
Use of protective gear	Yes	39	72.2	51	40.8
(Boots and gloves)	No	15	27.8	74	59.2
Handwashing with Soap	Yes	43	79.6	123	98.4
	No	11	20.4	2	1.6
Deworming practices	Yes	22	40.7	87	69.6
	No	32	59.3	38	30.4
Last time deworming	Less than 3 months	11	50.0	45	51.7
	Between 4–6 months	7	31.8	24	27.6
	Between 7–12 months	0	0.0	10	11.5
	More than 12 months	4	18.2	4	4.6
	Don’t remember	0	0.0	1	1.1
Type of anthelminthic drug used	Traditional	5	22.7	3	3.4
	Orthodox	17	77.3	84	96.6

In Greater Accra, the majority of the urban farmers were adults, (40.8%, 51/125) falling within the 20–30 years age range. Additionally, more than half (54.4%, 68/125) had primary education as their highest educational attainment. Unlike Tamale, female participation in urban farming was observed, though minimal (1.6%, 2/125). Only 40.8% (51/125) of farmers used PPEs, such as boots and gloves, while working on their farms. However, nearly all (98.4%, 123/125) practised handwashing with soap after work, and 69.6% (87/125) had dewormed using orthodox anthelmintic drugs within the past 12 months. The primary irrigation water source for these farmers was surface water, predominantly sourced from open drains (53.6%, 67/125) (Table 3).

### 3.3 Potential risk factor assessment

To examine the associations between risk factors of STH and STH infections, binary logistic regression was performed, analysing the predictor variables (risk factors) against the outcome (STH infection). The analysis revealed a strong association between the non-usage of PPE and STH infection (Odd Ratio (OR) 4.3, 95% Cl, 1.03–18.00, *p*-value = 0.04). However, no significant associations were found with other predictors, such as handwashing (*p*-value = 0.99), farmers’ education level (*p*-value = 0.99), or deworming status (*p-*value = 0.65) (Table 4).

**Table 4:** Logistic regression of risk factors for STH infections among urban vegetable farmers (November 2020 – October, 2021).

Characteristics	OR	*P*- value	95% Cl
**Deworming practice**			
No	Ref		
Yes	0.73	0.65	0.19–2.82
**PPE usage for Irrigation**			
No	Ref		
Yes	4.31	0.04	1.03–18.00
**Handwashing with soap**			
No	Ref		
Yes	0.00	0.99	–
**Education**			
No formal education	Ref		
Primary Education	80.00	0.99	–
JHS	90.00	0.99	–
SHS/ A Level	0.933	1.00	–
University	0.858	1.00	–
Informal education	75.00	0.99	–
**Water Source**			
Pipe water	–	0.91	–
Surface water/Open drain	0.46	0.51	0.04–4.72
Hand dug wells	0.85	0.87	0.12–6.17
Others	0.62	0.68	0.06–5.88

*OR = Odd ratio

## 4. Discussion

The findings of this study are highly significant, as it included urban farmers from multiple vegetable farm sites across different geographical regions (Northern-Tamale and Southern-Greater Accra regions), offering broader surveillance. Furthermore, the study utilized molecular diagnostic techniques (QPCR) to detect STHs in the stool samples of urban vegetable farmers in Ghana, providing more up-to-date and accurate data on the prevalence of STH infections among this population.

The *ITS1* and *18S* genes were the most amplified genes in the stool samples of the farmers, identifying roundworm (*A. lumbricoides)* and hookworm (*A. duodenale)* parasites. These findings were also consistent with previous studies in Kumasi [16], Abia state [27] and Kaduna States [28] in Nigeria, as well as in Tanzania [29]. This underscores the risk faced by urban vegetable farmers in acquiring helminthiasis, which is closely associated with diverse health outcomes, nutritional deficiencies, and reduced productivity among those infected [30] Additionally, infected farmers could act as carriers and potential sources of parasite transmission within the urban population [31].

However, the observed prevalence rates were relatively low, supporting the declining trend of STH infections both in Ghana and globally. This decline is attributed to the implementation of preventive chemotherapy with anthelmintic interventions being targeting STHs infections since 2001 [6,7,32].

This study found that the majority of farmers engaged in urban vegetable production were adult male within their economically active years, starting from the age of 20 years. This finding is consistent with reports from vegetable production studies in Oyo State in Nigeria [33], South Africa [34], Greater Accra and Wa municipality in Ghana respectively [35,36] This suggests that adult males involved in vegetable farming may be at a higher risk of STH infections.

This study has revealed that the lack of PPE usage among farmers during their work significantly increased their risk of STH infection compared to other risk factors. Many of these farmers often work and commute across the farms barefoot, without basic protective equipment such as boots and gloves, aligning with findings from previous studies [3,27]. The lack of PPEs contributed to the prevalence of hookworm and *S. stercoralis* among the urban farmers in Greater Accra, where PPEs usage was notably low. These parasites infect their hosts either through skin penetration or oral ingestion, often facilitated by the attachment of eggs to fingernails [37,38]. The STH prevalence observed among vegetable farmers in Greater Accra could be linked to their frequent contact with irrigation water sourced primarily from open drains, which were the most commonly used water sources [10,11,39]. These open drains are susceptible to contamination through practices such as open defecation on farmlands, a consequence of the lack of toilet facilities, and improper disposal of refuse, both of which are significant contributors to the presence of STH egg [3] Open defecation and poorly located refuse dumps near open drains can further elevate STH levels in irrigation water through runoff. In contrast, the absence of STH infections among farmers in Tamale could be attributed to the use of improved irrigation water sources, which are recognized as effective interventions for reducing STH infections [40,41], as well as the consistent use of PPE during farm activities.

This study highlights several policy and practice implications. Firstly, Agricultural Extension Officers (AEOs) assigned to these vegetable farms should intensify education on the importance of using basic protective gear, such as gloves and boots, to minimize direct contact with contaminated irrigation water and soil. Additionally, promoting good agricultural practices should be prioritized to reduce the risk of infections among farmers during their activities. Lastly, existing regulation (by-law) prohibiting the use of water from open drains, often contaminated with untreated wastewater, in urban vegetable production should be strictly enforced. Advocacy and interventions encouraging the adoption of improved water sources for irrigation in urban vegetable farming should also be strengthened.

A key limitation of this study was the reluctance of farmers to provide stool samples for analysis, primarily due to superstitious beliefs and a lack of feedback from previous research. However, they were reassured that the study could generate results that would be shared with them to help improve their farming practices.

## 5. Conclusion

The findings of this study reveal a low prevalence of STH infections among urban vegetable farmers in Greater Accra and no prevalence in Tamale, with lack of PPE usage being identified as the highest risk factor for STH infections. These results highlight the need for action from the Ministry of Food and Agriculture and other stakeholders involved in farmers’ health and urban food security. Interventions, including training on good farming practices, health education, proper sanitation, and the use of basic PPEs, should be strengthened through the assigned AEOs. This is crucial as the health of urban vegetable farmers directly impacts urban food security and the nutritional status of city residents. Additionally, molecular techniques should be prioritized for detecting STHs in areas with low prevalence and among those using anthelmintics, given their high specificity and sensitivity.

## Supporting information

S1 File Supplementary information. (ZIP)
