# Supplementary material for: Prevalence and determinants of Soil-Transmitted Helminths among urban vegetable farmers in Ghana
Source: PLoS One. 2025 May 15;20(5):e0323486. doi: 10.1371/journal.pone.0323486 (PMC12080784; doi:10.1371/journal.pone.0323486)
Supplement: S1 File — (ZIP) [file pone.0323486.s001.zip › Ethical clearance I.pdf]

KWAME NKRUMAH UNIVERSITY OF SCIENCE AND TECHNOLOGY
COLLEGE OF HEALTH SCIENCES
SCHOOL OF MEDICAL SCIENCES / KOMFO ANOKYE TEACHING HOSPITAL
COMMITTEE ON HUMAN RESEARCH, PUBLICATION AND ETHICS

Our Ref: CHRPE/AP/645/19

5th November, 2019.

Mr. Gerard Quarcoo
Department of Theoretical
and Applied Biology
KNUST-KUMASI.

Dear Sir,

LETTER OF APPROVAL

Protocol Title: ***"Pathogenic Microbes and Endocrine Disrupting Chemicals in Wastewater Used in Urban Agriculture in the Northern and Greater Accra Regions of Ghana."***

Proposed Site: ***Biomedical and Public Health Research Unit (CSIR – Water Research Institute, Accra).***

Sponsor: ***Principal Investigator.***

Your submission to the Committee on Human Research, Publications and Ethics on the above-named protocol refers.

The Committee reviewed the following documents:

- A notification letter of 9th October, 2019 from the Water Research Institute, Accra, Ghana (study site) indicating approval for the conduct of the study at the Institution.
- A Completed CHRPE Application Form.
- Participant Information Leaflet and Consent Form.
- Research Protocol.
- Questionnaire.

The Committee has considered the ethical merit of your submission and approved the protocol. The approval is for a fixed period of one year, beginning 5th November, 2019 to 4th November, 2020 renewable thereafter. The Committee may however, suspend or withdraw ethical approval at any time if your study is found to contravene the approved protocol.

Data gathered for the study should be used for the approved purposes only. Permission should be sought from the Committee if any amendment to the protocol or use, other than submitted, is made of your research data.

The Committee should be notified of the actual start date of the project and would expect a report on your study, annually or at the close of the project, whichever one comes first. It should also be informed of any publication arising from the study.

Thank you, Sir, for your application.

Yours faithfully,

Rev. Prof. John Appiah-Poku.
Honorary Secretary
FOR: CHAIRMAN

Room 7 Block J, School of Medical Sciences, KNUST, University Post Office, Kumasi, Ghana
Phone: +233 3220 63248 Mobile: +233 20 5453785 Email: chrpe.knust.kath@gmail.com / chrpe@knust.edu.gh

Scanned with CamScanner
