# Supplementary figures and images for: Prevalence and determinants of Soil-Transmitted Helminths among urban vegetable farmers in Ghana
Source: PLoS One. 2025 May 15;20(5):e0323486. doi: 10.1371/journal.pone.0323486 (PMC12080784; doi:10.1371/journal.pone.0323486)

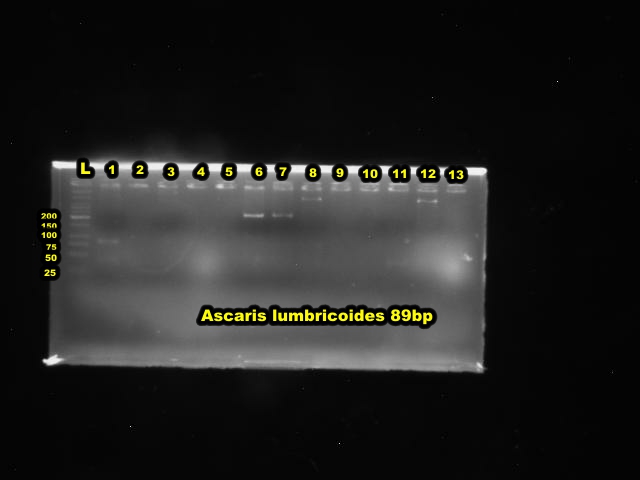

Supplement: S1 File — (ZIP) [file pone.0323486.s001.zip › Labelled gel images of STHs in stools of Farmer group two/Ascaris 1-13.jpg]

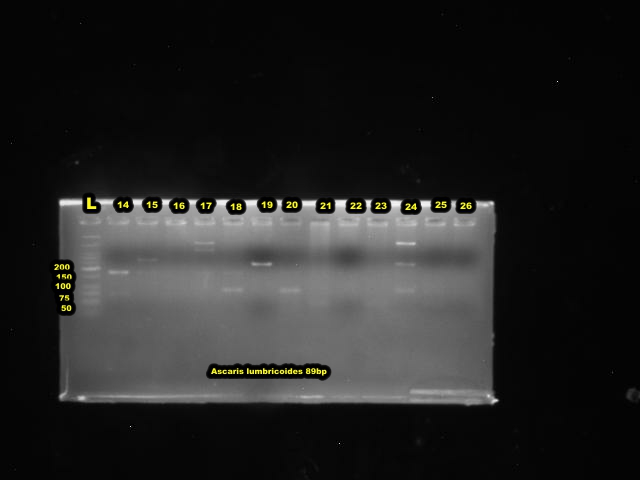

Supplement: S1 File — (ZIP) [file pone.0323486.s001.zip › Labelled gel images of STHs in stools of Farmer group two/Ascaris 14-26.jpg]

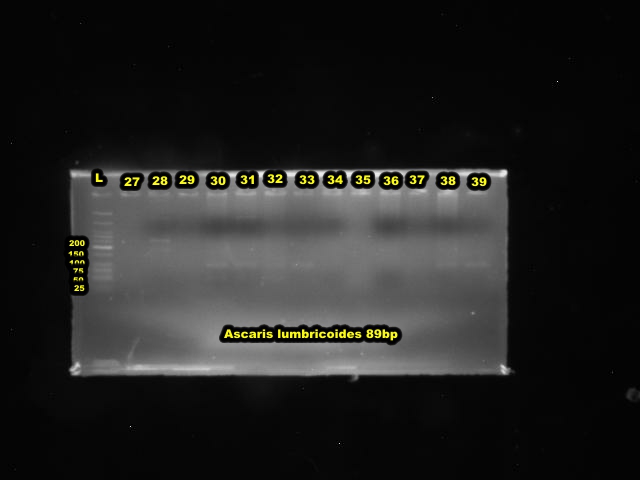

Supplement: S1 File — (ZIP) [file pone.0323486.s001.zip › Labelled gel images of STHs in stools of Farmer group two/Ascaris 27-39.jpg]

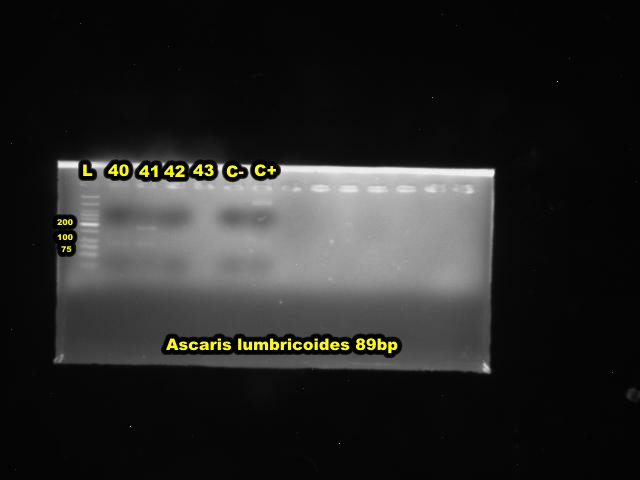

Supplement: S1 File — (ZIP) [file pone.0323486.s001.zip › Labelled gel images of STHs in stools of Farmer group two/Ascaris 39-43.jpg]

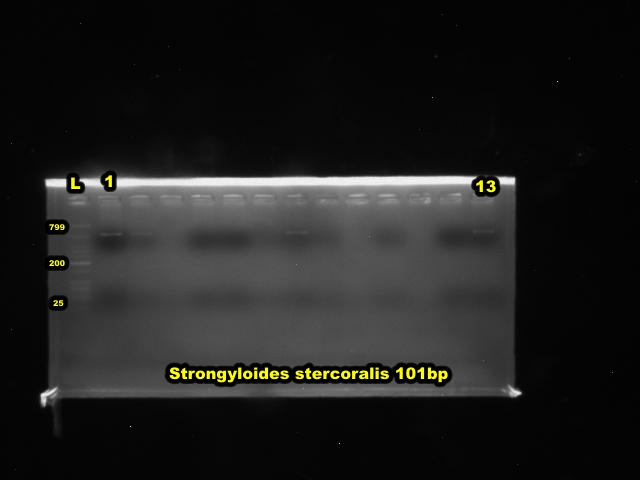

Supplement: S1 File — (ZIP) [file pone.0323486.s001.zip › Labelled gel images of STHs in stools of Farmer group two/STRO 1-13.jpg]

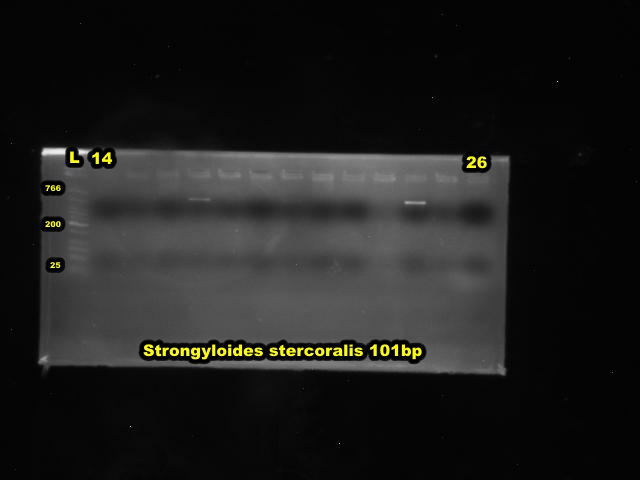

Supplement: S1 File — (ZIP) [file pone.0323486.s001.zip › Labelled gel images of STHs in stools of Farmer group two/STRO 14-26.jpg]

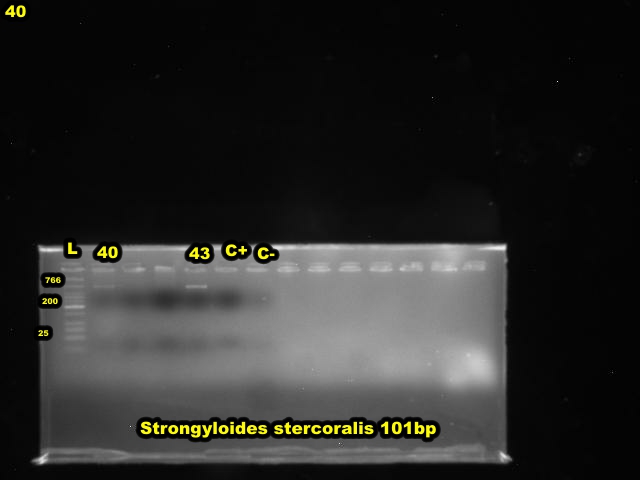

Supplement: S1 File — (ZIP) [file pone.0323486.s001.zip › Labelled gel images of STHs in stools of Farmer group two/STRO 40-43.jpg]

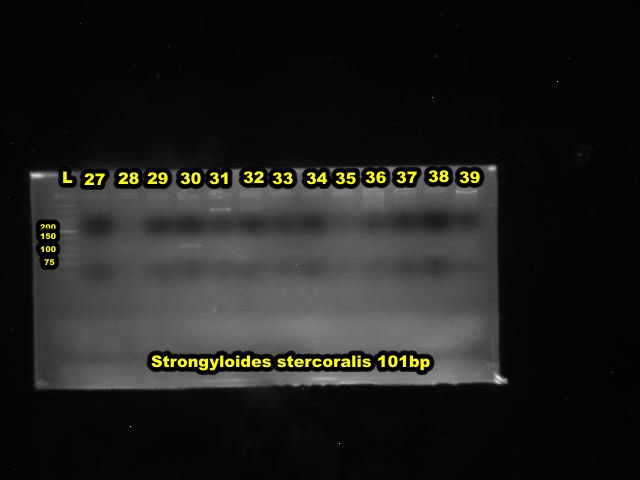

Supplement: S1 File — (ZIP) [file pone.0323486.s001.zip › Labelled gel images of STHs in stools of Farmer group two/Strongly 27-39.jpg]
