# Supplementary material for: Prevalence and determinants of Soil-Transmitted Helminths among urban vegetable farmers in Ghana
Source: PLoS One. 2025 May 15;20(5):e0323486. doi: 10.1371/journal.pone.0323486 (PMC12080784; doi:10.1371/journal.pone.0323486)
Supplement: S1 File — (ZIP) [file pone.0323486.s001.zip › Informed consent Template.doc]

**Participant Information Leaflet and Consent Form**

**This leaflet must be given to all prospective participants to enable them know enough about the research before deciding to or not to participate**

**Title of Research:**

(For example: Phase III randomized clinical trial of “New Toothpaste” for Treatment of Fetororis)

**Name(s) and affiliation(s) of researcher(s):**

For example: This study is being conducted by Dr. L. Mensah of the Komfo Anokye Teaching Hospital, Kumasi and Dr. K.O. Appiah of the University of Development Studies, Tamale)

**Background (Please explain simply and briefly what the study is about):**

**Purpose(s) of research:**

(For example: The purpose of this research is to find out whether “New Toothpaste” is better than the current best mouth washing practice in reducing mouth odour)

**Procedure of the research, what shall be required of each participant and approximate total number of participants that would be involved in the research:**

(For example: We will use lottery to divide participants in this study into 2 groups. Each participant will receive a year’s supply of toothpaste and 12 toothbrushes. Neither you nor your doctors will know whether you have received the “New Toothpaste”or the Old toothpaste. You will be required to brush your teeth for 2 minutes every morning with the toothpaste that you have been given. You should not use any other mouth cleaning product during the course of this trial. Once a month, you will be required to come to the research centre where the doctors will examine your teeth and take measurements of your mouth odour using an odourmeter. In total we expect to recruit 100 participants into this study throughout the country.)

**Risk(s):**

(For example: The “New Toothpaste” used in this study may cause some gum swelling, itching, discoloration and toothache. Some of your teeth may fall out reducing your ability to chew certain foods. You may develop burning sensation in the mouth, loss of appetite and stomach discomfort. We do not know how this toothpaste will affect the unborn child so it is advisable that you do not become pregnant during the course of your participation in this trial. In the event that you become pregnant, you should tell your doctor so that you can be withdrawn from this study.)

**Benefit(s):**

(For example: The goal of this research is to find ways of reducing mouth odour. We hope that the “New Toothpaste” will reduce mouth odour and make your teeth whiter and brighter but we cannot guarantee/be certain about this; this is research.)

**Confidentiality:**

(For example: All information collected in this study will be given code numbers. No name will be recorded. Data collected cannot be linked to you in anyway. No name or identifier will be used in any publication or reports from this study. However, as part of our responsibility to conduct this research properly, we may allow officials from the Food and Drugs Board (for clinical trials) and ethics committees to have access to your records.)

**Voluntariness:**

(For example: Taking part in this study should be out of your own free will. You are not under obligation to. Research is entirely voluntary.)

**Alternatives to participation:**

(For example: If you choose not to participate, this will not affect your treatment in this hospital/institution in any way.)

**Withdrawal from the research:**

(For example: You may choose to withdraw from the research at anytime without having to explain yourself. You may also choose not to answer any question you find uncomfortable or private).

**Consequence of Withdrawal:**       (For example: There will be no consequence, loss of benefit or care to you if you choose to withdraw from the study. Please note however, that some of the information that may have been obtained from you without identifiers (name etc), before you chose to withdraw, may have been modified or used in analysis reports and publications. These cannot be removed anymore. We do promise to make good faith effort to comply with your wishes as much as practicable.)

**Costs/Compensation:**       (For example: For your time/inconvenience/transport to the hospital, we will compensate you with GH¢2.00 to show our appreciation for your participation).

**Contacts:** (For example: If you have any question concerning this study, please do not hesitate to contact Dr. Mensah (Name of Researcher or PI) on 025 5552255).

**Further, if you have any concern about the conduct of this study, your welfare or your rights as a research participant, you may contact:**

**The Office of the Chairman**

**Committee on Human Research and Publication Ethics**

**Kumasi**

**Tel: 03220 63248 or 020 5453785**

**CONSENT FORM**

**Statement of person obtaining informed consent:**

I have fully explained this research to ____________________________________ and have given sufficient information about the study, including that on procedures, risks and benefits, to enable the prospective participant make an informed decision to or not to participate.

DATE: _____________________	NAME: _________________________________

**Statement of person giving consent:**

I have read the information on this study/research or have had it translated into a language I understand. I have also talked it over with the interviewer to my satisfaction.

I understand that my participation is voluntary (not compulsory).

I know enough about the purpose, methods, risks and benefits of the research study to decide that I want to take part in it.

I understand that I may freely stop being part of this study at any time without having to explain myself.

I have received a copy of this information leaflet and consent form to keep for myself.

NAME:_________________________________________________________________

DATE: ____________	SIGNATURE/THUMB PRINT: ___________________

**Statement of person witnessing consent (Process for Non-Literate Participants):**

I	(Name of Witness) certify that information given to

(Name of Participant), in the local language, is a true reflection of what l have read from the study Participant Information Leaflet, attached.

WITNESS’ SIGNATURE (maintain if participant is non-literate): ____________________

MOTHER’S SIGNATURE (maintain if participant is under 18 years): ________________

MOTHER’S NAME: ______________________________________________________

FATHER’S SIGNATURE (maintain if participant is under 18 years): _________________

FATHER’S NAME: ______________________________________________________
